# Using COVID-19 Symbols in Anti-Smoking Fear Appeal Advertisements for Encouraging Smoking Cessation among Israeli Smokers

**DOI:** 10.3390/ijerph182010839

**Published:** 2021-10-15

**Authors:** Iris Gavish, Yossi Gavish

**Affiliations:** Faculty of Business Administration, Ono Academic College, Kiryat Ono 5545173, Israel; Yg@ono.ac.il

**Keywords:** fear appeals, threatening message, health behavior, COVID-19, anti-smoking advertisements

## Abstract

The current study aims to reveal whether using COVID-19 as the threatening message in anti-smoking ads will influence smokers differently than other threat appeals. All ads that were chosen for this study were created by the Israel Cancer Association/the Israeli Ministry of Health. Since the coronavirus has proven to have far-reaching effects on the human respiratory system, it is directly connected to smoking. The present study included semi-structured in-depth interviews with experts, a pre-test (*n* = 106) and an online questionnaire including 721 participants (adults aged 18–30 versus 55+). The findings indicated that when using the COVID-19 symbol as the threatening message in an anti-smoking ad for the older participants, smoking cessation intentions were higher than when using a cigarette simulating a ‘gunpoint’ threat (*p* < 0.08). Additionally, when using the COVID-19 symbol, there was a positive relation between participants’ age and smoking cessation intentions. The average smoking cessation intention for the participants from the older age group (*M* = 3.05, *SD* = 1.07) was higher than the average for the participants from the young age group (*M* = 2.80, *SD* = 1.13). Finally, when using impotence (for men) and pregnancy risks (for women) as the threatening message in the ads for young respondents, smoking cessation intentions were higher than when using COVID-19 (*p* < 0.05). The results may help decision-makers and public health officials in choosing the marketing communication suited for conveying messages aimed to encourage people to reduce/quit smoking.

## 1. Introduction

In late 2019, severe acute respiratory syndrome coronavirus 2 (SARS-CoV-2) was discovered in Wuhan, China, causing the coronavirus disease 2019 (COVID-19) [1,2]. On 11 March 2020, the World Health Organization (WHO) formally declared COVID-19 to be a global pandemic. According to WHO, the COVID-19 death toll worldwide was 4,390,467 as of 19 August 2021, and it has been rising daily.

COVID-19 manifestations are extremely diverse [1], with the majority of infected individuals suffering from mild symptoms or having no symptoms at all [3]. Mortality rates occur predominantly in the subgroup of patients who have severe respiratory failure. The major entrance ways of the virus are through mucosal tissues: the mouth, nose, and upper respiratory tract [4].

The consequences of tobacco smoke exposure include inflammatory processes in the lung, increased mucosal inflammation, increased permeability in epithelial cells, mucus overproduction, and impaired mucociliary clearance [5]. Smokers are not only considered to be an at-risk group for severe COVID-19 infection, but are also considered to be possible transmitters of the virus for both active and passive smokers [6]. Therefore, it is important to gain knowledge about host factors, particularly avoidable host factors such as smoking, in order to reduce virus-related damage and the severity of the disease [4].

According to studies, one of the most prevalent appeals used in anti-smoking advertisements is fear appeal [7]. Fear appeals are persuasive messages intended to provoke fear by describing the negative consequences that individuals will suffer if they do not stop risky behaviors and/or carry out preventive behaviors [8]. Fear appeals are often used in programs such as AIDS prevention [9] and anti-smoking advertising [10]. Naturally, the fear and uncertainty surrounding the COVID-19 pandemic is in the media focus, triggering one of the most monumental, worldwide social and health care crises.

However, smoking remains a severe problem globally. Data show that 8000 people die in Israel every year both due to active and passive smoking. In the last decade about 80,000 people died in Israel due to smoking damages [11].

Furthermore, there are other severe risks associated with smoking. Studies show that maternal smoking during pregnancy doubled the risk for sudden unexpected infant death [12], and that smoking during pregnancy reduces infants’ mean birth weight by 320 g in infants whose mothers smoked between 6 and 10 cigarettes per day, and by 435 g in infants whose mothers smoked between 11 and 40 cigarettes per day [13]. Regarding men, studies revealed that impotence is directly proportional to smoking, and that quitting smoking improves male erectile function in all age groups between the ages of 30–60 years [14,15].

Since we aimed to compare between genders, we focused on the outcomes of smoking during pregnancy and impotence when talking about the severe risks associated with smoking for the young participants. Therefore, we chose risks that are specifically associated to each gender.

On the one hand, due to the emergence of the COVID-19, people worldwide were subjected to radical changes in their daily routine, varying from social distancing and stay-at-home policies to a total quarantine. Such changes, as well as the associated economic stress, may have a psychological impact that might even lead people to begin smoking or increase the number of cigarettes they smoke per day [16]. On the other hand, the fear surrounding the new virus bearing a higher threat to smokers may encourage people to quit smoking. An Italian survey that was conducted during the lockdown period declared a minor decrease of cigarette consumption [17]. The current study aims to reveal whether using a COVID-19 fear appeal in anti-smoking ads will affect smokers differently than anti-smoking ads using other fear appeals. Moreover, our study aims to estimate whether anti-smoking ads using the COVID-19 fear appeal will influence the older smokers (55 years old or above) more profoundly, since they are considered a high-risk group. Finally, we investigate whether COVID-19 fear appeals in advertisements will have a robust influence, adding to the natural effect of the COVID-19 public discourse. Therefore, despite the contrasting effects that were mentioned above, we think that since the COVID-19 outbreak is in the media focus around the world, this may be a great opportunity to leverage the pandemic and harness the anxiety it causes to better fight the war against smoking. To the best of our knowledge, no previous studies have compared the impact of the COVID-19 fear appeal in anti-smoking ads to other fear appeals among young and old smokers.

### 1.1. The Impact of COVID-19 on the Elderly Population

COVID-19 poses a higher risk to the elderly population due to their frail immune system as well as the prevalence of chronic underlying diseases. This population is usually more severely infected, resulting in a higher death toll among the seniors and those with chronic underlying diseases [18]. Furthermore, a recent study noted that the emotional reaction of seniors over 60 years old to the COVID-19 outbreak is more significant than among the younger generations [19]. It was argued that the seniors are not only faced with the disadvantages caused by their physical condition, but are also affected mentally, experiencing depression and anxiety. Therefore, living during the COVID-19 crisis can accumulate stress and fear among the elderly [18].

Although the pathogenesis of severe COVID-19 and the associated respiratory failure is not quite clear, the association between higher mortality with older age is consistent [1]. Particularly, the respiratory system undergoes various physiological, immunological, and anatomical changes with age. The lung reaches maturity by the age of 20–25 years. Afterwards, aging is associated with ongoing decline in lung function [4,20].

### 1.2. Using Fear Appeals in Advertising

Fear is an emotional reaction to a threat which expresses some kind of danger. In most cases, fear has a significant effect on individual’s behavior, leading them to attempt to eliminate or deal with a threat or danger [21,22]. Studies have revealed that fear appeals effectively motivate behavior change. Specifically, it was noted that anti-smoking ads depicting the serious health consequences of smoking such as lung cancer by evoking fear are perceived as effective by both adults and youths [23,24,25].

Furthermore, the ordered protection motivation model (OPM) identified that emotional processes are linked indirectly to behavioral intentions [22]. According to the OPM model, two dimensions should be considered in creating fear appeals: threat appraisal and coping appraisal. Finally, fear appeal messages involve both cognitive and emotional processes, and both social threats and physical threats may lead to protection motivation.

The results of a recent study revealed that the use of fear appeal imagery in advertising activates brain regions associated with threat detection and contingency awareness, forming emotional appraisal [26].

The main objective of this study is to examine the use of COVID-19 imagery (symbol/face mask) in fear appeal ads as a tool for encouraging adults to quit smoking. In addition, the study aims to compare different types of anti-smoking fear appeal ads and their impact on different segments of the population (genders, age groups).

The uniqueness of the current study is the integration of anti-smoking fear appeal literature with the literature that deals with COVID-19 and its ramifications on human respiratory system. Moreover, this study compares between anti-smoking fear appeal ads using pregnancy risks, impotence, and the coronavirus risks regarding their effect on individuals’ smoking cessation intentions.

To the best of our knowledge, no studies regarding the use of COVID-19 in anti-smoking fear appeal ads have been published to date.

Based on the literature review our hypotheses are as follows:

**Hypothesis** **1** **(H1).** *When using COVID-19 as the threatening message in an anti-smoking ad for the older participants (aged 55 or above), smoking cessation intentions are higher than when using a threatening message which is not related to COVID-19.*

**Hypothesis** **2** **(H2).** *When using COVID-19 as the threatening message in an anti-smoking ad, there is a positive relation between participants’ age and smoking cessation intentions.*

**Hypothesis** **3** **(H3a).** *When using impotence as the threatening message in an anti-smoking ad for young males (aged 18–30), smoking cessation intentions are higher than when using COVID-19 as the threatening message.*

**Hypothesis** **3** **(H3b).** *When using pregnancy risks as the threatening message in an anti-smoking ad for young females (aged 18–30), smoking cessation intentions are higher than when using COVID-19 as the threatening message.*

Figure S2 illustrates the research process based on the literature reviewed, in-depth interviews and the pretest (see Figure S2).

## 2. Method

### 2.1. Sampling, Data Collection and Procedures

Data was collected in three stages: 

Stage 1: Semi-structured in-depth interviews with eight specialized lung experts from Israeli hospitals were conducted. The interviews included structured questions as well as listening to each expert’s opinions and comments regarding the relevant subjects from their perspectives as experts who treat smokers and lung disease patients. The purpose of this stage was to examine whether the intended direction of the research is consistent with the opinions of experts in the field based on their knowledge and experience. All the data from the interviews were organized and compared. This stage was used as an exploratory phase to gain a better understanding of the problem and acquire confirmation to pass to the quantitative stage of the research.

Stage 2: A pre-test including 106 adult smokers was conducted in order to test the reliability of the research scales.

Stage 3: The main study included 721 adult smokers. “Smokers” were respondents who defined themselves as such in a separate question and answered that they smoke at least a cigarette a day. The question: ‘do you usually smoke?’ was presented as a screening question, and only those who answered positively remained part of the sample and continued to the next question.

In stages 2 and 3, data were collected through online self-administered questionnaire with three scales and demographic items. The questionnaires were distributed online by “I panel- The Israeli Online Panel” among a sample of smoking adults from different regions in Israel in order to obtain a good representation of the whole population. The survey was conducted during the last quarter of 2020.

The sample was divided into two age sub-groups. Group 1 included 418 adult smokers between the ages of 18 and 30. Group 2 included 303 adult smokers aged 55 or above. The age groups were selected in accordance with the experts’ opinions from the in-depth interviews. Since we aimed to differentiate between participants who belong to a segment in the population that is deeply affected by the virus (whom we assumed will be more likely to positively respond to the COVID-19 threats in the ads) and the much younger population, for whom the threat of becoming severely ill is minor (whom we assumed will be less likely to positively respond to the COVID-19 threats in the ads), we chose to focus our study on these margin age groups. Therefore, group 2 was chosen to represent smokers who are at high risk of suffering from severe COVID-19 complications according to the experts’ opinions. Each respondent was presented with an anti-smoking ad. Participants within different sub-groups were presented with different ads—all fear appeals, but only two with a COVID-19 threatening message. In order to validate the results, two types of COVID-19 fear appeal ads were used. All ads that were used for the study were created by the Israel Cancer Association and/or The Israeli Ministry of Health. Therefore, the connections presented in these ads (i.e., between smoking and COVID-19, as well as between smoking and pregnancy risks or impotence) were established by professional authorities. The questionnaire and the in-depth interview formats are presented in supplementary files S1 and S3. Table 1 provides a description of the sample demographics.

### 2.2. Measures

In stage 1, we conducted structured in-depth interviews (see Supplementary File 1). All interviewees were specialists in internal medicine, specifically in lung diseases. Most of them were directors and head of departments of lung units in different public Israeli hospitals, and the rest were deputy directors. Table S4 and Supplementary Material S5 include the expert’s opinions, as well as some of the statements that surfaced from the in-depth interviews. Based on the in-depth interviews, we proceeded to the pre-test stage.

A pre-test including 106 adult smokers was conducted in order to test the reliability of the research scales. According to the pre-test, all the study’s scales met the required standard of the reliability (Cronbach’s alpha above 0.7), enabling us to proceed to the main study.

#### 2.2.1. Procedure and Scales

In order to test the study’s hypotheses, we used the following scales:

‘Intentions to quit smoking’ were measured using the Intentions to Quit Smoking Scale [27], which is a 3-item Likert scale. Cronbach’s alpha for our pre-test sample was 0.82, which exhibits a satisfactory internal consistency reliability.

‘The perceived threatening message scale’ was measured using the Perceived Threatening Message Scale [27], which is a 3-item Likert scale. Cronbach’s alpha for our pre-test sample was 0.93, which exhibits a satisfactory internal consistency reliability.

‘Attitudes towards smoking’ were measured using the Attitudes Towards Smoking Scale (ATS-18) [28], which is an 18-item scale. Cronbach’s alpha for our pre-test sample was 0.83, which exhibits a satisfactory internal consistency reliability.

As all scales were originally in English, a conventional back-translation technique was used. One bilingual individual translated the original questionnaire to Hebrew. A second bilingual individual, blind to the original questionnaire, back-translated the Hebrew version into English. Then, the two translators and one of the authors evaluated the translations for wording, content, and local applicability and equivalence. Minor disagreements were resolved in this stage, leading to the final version.

Table S6 presents scales’ reliabilities. Table S7 presents scales’ items and authors, and Table S8 presents the main study sub-groups (See Supplementary Materials).

#### 2.2.2. Study’s Variables

##### Dependent Variable

Intentions to quit smoking were measured using the Intentions to Quit Smoking Scale [27], which is a 3-item Likert scale. Cronbach’s alpha for our main study’s sample was 0.82, which exhibits satisfactory internal consistency reliability.

##### Independent Variables

1. The perceived threatening message scale was measured using the Perceived Threatening Message Scale [27], which is a 3-item Likert scale. Cronbach’s alpha for our main study’s sample was 0.94, which also exhibits satisfactory internal consistency reliability;
2. Attitudes towards smoking were measured using the Attitudes Towards Smoking Scale (ATS-18) [28], which is an 18-item scale. Cronbach’s alpha for our main study’s sample was 0.80, which also exhibits satisfactory internal consistency reliability;
3. Demographic variables (see Table 1 above and Supplementary Material S3).
4. Cigarette dependence (measured by the number of cigarettes per day).

## 3. Results

We divided the statistical analysis of the main study into two stages: regressions and *t*-tests, all of which were conducted using IBM- SPSS Statistics version 25.

### 3.1. Regressions

We conducted a regression analysis which included seven separate linear regressions according to the different advertisements that were presented to the respondents. The dependent variable was the Intentions to Quit Smoking Scale, and the independent variables included two scales: the Perceived Threatening Message Scale and the Attitude Towards Smoking Scale. The independent variables also included the number of cigarettes per day and demographics variables.

The findings presented in Table 2 reveal the following:

1. All regressions were significant at the highest level (0.00);
2. The variable ‘perceived threatening message’ was significant in all regressions;
3. The variable ‘attitude towards smoking’ (independent variable) was significant in most regressions (not significant only in regression number 6—young women—Corona);
4. The variable ‘number of cigarettes per day’ was significant for young women with a negative coefficient (in both threat appeal messages—Corona/pregnancy). We assume that women who smoke less cigarettes per day are less addicted to cigarettes. Therefore, when they were exposed to a threatening message, they demonstrated higher levels of intentions to quit smoking.

### 3.2. Analyzing the Hypotheses

We tested the study’s hypotheses using a between groups *T*-test analysis for each hypothesis, as follows:

Hypothesis 1 (H1)

The analysis of the independent samples T-test indicated that there was no significant difference in the intentions to quit smoking between the older participants who were presented with an anti-smoking ad that depicted a COVID-19 threatening message (symbol/face mask) and those who were presented with an anti-smoking ad simulating a ‘gunpoint’ threat (*t* (301) = 0.50, *p* > 0.10). Therefore, we decided to examine each COVID-19 message separately.

For the coronavirus symbol, the results revealed a significant difference in the intentions to quit smoking between the older participants who were presented with an anti-smoking ad that depicted a COVID-19 threatening message and those who were presented with an anti-smoking ad simulating a ‘gunpoint’ threat (*t* (201) = 1.42, *p* = 0.079). The average smoking cessation intention for the participants from the COVID-19 group (M = 3.05, SD = 1.07) was higher than the average for the participants from the ‘gunpoint’ group (M = 2.83, SD = 1.09).

However, for the coronavirus mask, the difference was not statistically significant (*t* (203) = −0.50, *p* = 0.201). Moreover, the average smoking cessation intention for the participants from the COVID-19 group (*M* = 2.76, *SD* = 1.16) was lower than the average for the participants from the ‘gunpoint’ group (*M* = 2.83, *SD* = 1.09). Thus, hypothesis H_1_ was confirmed only for the anti-smoking ad which presented the COVID-19 symbol. We conclude that, when choosing an anti-smoking threat appeal, it is crucial to choose not only the message but also the way in which it will be depicted on the ad.

Hypothesis 2 (H2)

Similar to the case of the first hypothesis, the analysis of the independent samples T-test indicated that there was no significant difference in the intentions to quit smoking between the two age groups who were presented with an anti-smoking ad that depicted a COVID-19 threatening message *(t* (404) = −1.05, *p* > 0.10). Therefore, we decided to examine each COVID-19 message (symbol/face mask) separately.

For the coronavirus symbol, the results revealed a significant difference in the intentions to quit smoking between the two age groups’ participants (*t* (200) = −1.63, *p* = 0.050). The average smoking cessation intentions for the participants from the older age group (*M* = 3.05, *SD* = 1.07) was higher than the average for the participants from the young age group (*M* = 2.80, *SD* = 1.13).

Nevertheless, for the coronavirus mask, the difference was not statistically significant (*t* (202) = 0.08, *p* > 0.10). As expected from the results, the average smoking cessation intention for the participants from the older age group (*M* = 2.76, *SD* = 1.16), was almost similar to the average for the participants from the young age group (*M* = 2.77, *SD* = 1.20). Thus, hypothesis H_2_ was also confirmed only for the anti-smoking ad which presented the COVID-19 symbol. This finding is in line with the literature reviewed, implying that using COVID-19 as the threatening message in the anti-smoking ad will be effective among the oldest population, which is a high-risk group, compared to the young population.

Hypothesis 3 (H3a)

The analysis of the independent samples T-test indicated that there was a significant difference in the intentions to quit smoking between the young male respondents who were presented with an anti-smoking ad that depicted a COVID-19 threatening message, and those who were presented with an anti-smoking ad that depicted the impotence threat (*t* (207) = −1.67, *p* < 0.05). As we assumed, the average smoking cessation intention for the participants from the impotence group (*M* = 3.15, *SD* = 1.27) was higher than the average for the participants from the COVID-19 group (*M* = 2.39, *SD* = 1.26). Therefore, hypothesis H_3_a was confirmed for both COVID-19 threatening messages (symbol and face mask).

Hypothesis 3 (H3b)

The analysis of the independent samples T-test indicated that there was no significant difference in the intentions to quit smoking between the young female respondents who were presented with the anti-smoking ad that depicted a COVID-19 threatening message (symbol/face mask) and those who were presented with the anti-smoking ad that depicted the pregnancy risks threat (*t* (207) = −1.09, *p* > 0.10). Therefore, we decided to examine each COVID-19 message separately.

For the coronavirus symbol, the results showed no significant difference in the intentions to quit smoking between the female participants who were presented with the anti-smoking ad that depicted a COVID-19 threatening message and those who were presented with the anti-smoking ad that depicted a pregnancy risks threat (*t* (155) = 0.05, *p* > 0.10). The average smoking cessation intention for the participants from the COVID-19 group (*M* = 3.01, *SD* = 1.10) was similar to the average for the participants from the pregnancy risks group (*M* = 3.00, *SD* = 1.03).

However, for the coronavirus mask, the difference was statistically significant (*t* (155) = −1.83, *p* < 0.05). Moreover, the average smoking cessation intention for the participants from the COVID-19 group (*M* = 2.65, *SD* = 1.25) was lower than the average for the participants from the pregnancy risks group (*M* = 3.00, *SD* = 1.03). Hence, hypothesis H_3_b was confirmed only for the anti-smoking ad which presented the COVID-19 face mask.

We conclude that when choosing an anti-smoking threat appeal, it is crucial to choose the form of depicting the threatening message according to the market segment that the ad is targeted for (old/young/men/women).

## 4. Discussion

### 4.1. Theoretical Implications

Although the distribution of COVID-19 vaccines around the world is in progress, some issues regarding the vaccines remain a mystery. For example, according to Israeli data, COVID-19 vaccines provide protection for about six months. Afterward, a booster dosage of vaccine is needed. However, the FDA has authorized the booster only for adults aged 65 or above. Will other countries follow suit? Will the vaccines also provide enough protection against infecting others? How quickly could COVID-19 vaccines stop the pandemic? Will the vaccines protect people from new coronavirus variants that are spreading around the world? Insufficient information has been gathered about the vaccines’ implications and level of efficacy. In the meantime, the coronavirus pandemic is still rampant around the globe.

The current study, which dealt with the influence of using COVID-19 as the threatening message in anti-smoking ads, may be used as a case study for examining the deterrent potential inherent in a global medical mega-event, and be leveraged for reducing the consumption of cigarettes, which have negative effects on individuals as well as our society.

It was noted that more of a perceived threat generally leads to more fear, and that this is a significant effect in almost all groups [29]. Over the years, fear appeal has been used in advertising by utilizing the fear of cancer and other medical threats to encourage people to quit smoking. We hypothesized that the effect of using the COVID-19 symbol as the threatening message in anti-smoking advertising for the older population has greater potential than using other common medical fear appeal anti-smoking ads (such as cancer), partly because in cases of cancer or other diseases, the risk of developing the disease is not imminent, while the coronavirus poses an immediate danger. According to the in-depth interviews, a risk that is perceived as imminent is more effective than a potential future risk.

The research presented in this paper is a comprehensive study both in terms of the sample size and due to the combined use of qualitative and quantitative research. 

The first stage of the study included in-depth interviews with eight senior specialists in lung diseases, most of whom are directors and heads of departments of lung units in different public Israeli hospitals from diverse geographic areas. This procedure enabled us to get a geographic and demographic coverage that characterizes the Israeli population in terms of the socio-economic and demographic aspects of the mosaic of religions in Israel (in the northern State of Israel, there is a relatively high concentration of non-Jews). This qualitative study supported our research directions, and along with the literature review, formed the basis for the three hypotheses.

In the second stage of the study, a pre-test was conducted involving 106 adult smokers. In this stage the internal consistency reliability of each scale in the questionnaires was tested (Cronbach’s alpha). The findings of the pre-test supported the reliability of the scales, enabling us to proceed to the main study.

The main study included 721 adult smokers from 2 age groups (18–30, and 55 or above). The respondents were presented with a variety of fear appeal anti-smoking ads (two types included a COVID-19 threatening message—a symbol and a face mask—while the others included different threating messages depending on the respondent’s age group and/or gender). The findings indicate that using the COVID-19 symbol as the threatening message for the older age group is more effective than using a non-coronavirus threat (‘gunpoint’), which is in line with our hypothesis based on most experts’ opinions and the literature reviewed. Both the reports that the coronavirus causes a disease that damages the lungs and affects adults more severely [18] and the knowledge that cigarettes harm the lungs [30] have led adults to recognize that COVID-19 poses an immediate life threat. This has led them to reconsider their smoking habits and raised their smoking cessation intentions. In addition, the comparison between the age groups revealed that the ad that included the COVID-19 symbol was more effective among the older respondents than among the respondents from the younger age group. This finding also supported our hypothesis and is in line with the literature review [19].

Moreover, the results revealed that the ad that included the COVID-19 mask was less effective compared with the ad that included pregnancy risks (for young women) or impotence risks (for young men), which was also consistent with most experts’ opinions.

Finally, in order to examine whether the COVID-19 message can affect smoking cessation intentions, we included two types of COVID-19 fear appeal ads (symbol and face mask). According to the results, the COVID-19 symbol was more effective compared with the face mask. The significance of this outcome is that even if the decision-makers choose to use COVID-19 as the threatening message, they are still required to pick the ultimate way to convey the threat that will lead to optimal internalization. The findings of the current study show that although in both cases the conveyed message was that the coronavirus is more dangerous to smokers, there was a significant difference in terms of the internalization of the message in favor of the symbol over the mask. A possible explanation could be that in the ad that included the COVID-19 symbol, the coronavirus was presented directly, whereas in the ad with the face mask, the mask was perceived as a means to minimize the risk of contracting the disease, and the message of the coronavirus threat was indirectly conveyed to participants.

### 4.2. Managerial Implications

Smoking is one of the main causes of lung cancer and other diseases. Not only does the damages of smoking include public health aspects, but they also cause economic costs emanating from the need for health treatments and the workload on the public health system. Hence, there is a need to use every possible measure to encourage people to quit smoking, as well as to prevent youth from starting the negative habit of smoking.

Therefore, there is a need for a combined public policy that incorporates education and guidance and harnesses the media to promote anti-smoking messages.

The current study examined the use of advertising tools exploiting a global crisis for conveying messages aiming at decreasing the problem of smoking. Thus, its outcomes may help decision-makers and public health officials in terms of evaluating the effectiveness of the marketing communication suited for conveying the messages that aim to encourage people to reduce smoking, or even quit smoking.

### 4.3. Limitations and Future Research

Despite the scope of this study, it is not without limitations. First, as was explained in the method section, the rationale of the age groups choice for our study was to differentiate between participants who belong to a segment in the population that is deeply affected by the virus and the much younger population, for whom the threat of becoming severely ill is minor. However, considering that smoking addiction is a longitudinal process, and that people aged 31–54 are still at reproductive age, excluding smokers aged 31–54 may be considered a limitation. 

Moreover, it is common knowledge that prevention of smoking is easier than smoking cessation. Adolescents constitute a large section of those who start smoking. Therefore, future research could test whether exposing teenagers to anti-smoking advertisements with COVID-19 as the threatening message impacts their attitude towards smoking. It could be interesting to examine the changes of attitudes before and after the exposure to the ad using the Attitudes Towards Smoking Scale (ATS-18) [28].

Additionally, the current study dealt with smoking cessation intentions rather than actual smoking cessation. Therefore, it is quite possible that there is a significant gap between the two variables, and that smoking cessation intentions do not necessarily lead to actual smoking cessation. Future study regarding actual smoking cessation is recommended.

Furthermore, the current study was conducted in Israel. In order to generalize the findings, it might be beneficial to replicate the study in countries that differ from Israel in terms of culture, specifically with regard to individualism and uncertainty avoidance [31].

Lastly, studying the efficacy of anti-smoking COVID-19 fear appeal advertising with time is recommended. That is, does, as with the vaccines, its effect decrease, and people return to their previous attitudes and need a ‘booster’ of fear appeals to maintain the reduction of smoking?

## Figures and Tables

**Table 1:** Sample demographics (*n* = 721).

Variable	*n*	%
Age		
18–30	418	58
55+	303	42
Education		
Elementary or under	17	2.4
High school without matriculation certificate	101	14
High school with matriculation certificate	195	27
Post-secondary education with non-academic certification	158	21.9
B.A	194	26.9
M.A or higher	56	7.8
Income		
Much lower than the average	184	25.5
Below average	191	26.5
Average	195	27
Above average	123	17.1
Much higher than the average	28	3.9
Gender		
Male	309	42.9
Female	412	57.1

**Table 2:** The main study regressions’ results.

Regression	Threatening Message	Sample Size	R^2^ (Sig.)	Significant Variables (β, Sig.)
1	Coronavirus symbol Older population (55+)	98	0.28 (0.00)	Message (0.33, 0.00)
				Attitude (0.36, 0.00)
2	Coronavirus face mask Older population (55+)	100	0.44 (0.00)	Message (0.61, 0.00)
				Attitude (0.16, 0.05)
3	Non-Corona Older population (55+)	105	0.28 (0.00)	Message (0.27, 0.00)
				Attitude (0.31, 0.00)
				Gender (−0.18, 0.04)
4	Coronavirus Young men (18–30)	104	0.42 (0.00)	Message (0.50, 0.00)
				Attitude (0.25, 0.01)
5	Non-Corona: Impotence Young men (18–30)	105	0.37 (0.00)	Message (0.50, 0.00)
				Attitude (0.18, 0.06)
6	Coronavirus Young women (18–30)	104	0.35 (0.00)	Message (0.45, 0.00)
				Number of cigarettes per day (−0.29, 0.00)
7	Non-Corona: Pregnancy risks Young women (18–30)	105	0.30 (0.00)	Message (0.50, 0.00)
				Attitude (0.16, 0.06)
				Number of cigarettes per day (−0.27, 0.00)
Total		721		

## Data Availability

The data presented in this study are available on request from the corresponding author. The data are not publicly available due to privacy and ethical reasons.

## Supplementary Materials

The following are available online at https://www.mdpi.com/article/10.3390/ijerph182010839/s1, Supplementary Material S1: In–Depth Interview, Figure S2: Illustration of the research process, Supplementary Material S3: The Questionnaire, Table S4: Summary of Experts’ Opinions, Supplementary Material S5: Experts’ Opinions–Statements, Table S6: Scales’ Reliabilities (Cronbach’s α), Table S7: Scale Names, Authors, and Items, Table S8: The Main Study’s Sub-groups.
