# Supplementary material for: Using COVID-19 Symbols in Anti-Smoking Fear Appeal Advertisements for Encouraging Smoking Cessation among Israeli Smokers
Source: Int J Environ Res Public Health. 2021 Oct 15;18(20):10839. doi: 10.3390/ijerph182010839 (PMC8535395; doi:10.3390/ijerph182010839)
Supplement: Supplementary file 1 [file ijerph-18-10839-s001.zip › ijerph-1251298-supplementary.pdf]

Supplementary Materials

Supplementary Material S1: In–Depth Interview

Name: ______________________

Hospital / Clinic: _____________

Specialty: ___________________

In-depth Interview with a Specialist Doctor

1) In your opinion, to what extent does smoking increase the severity of the COVID-19 disease, as well as the risk of suffering from complications? __________ (from 1 representing "extremely low" to 5 representing "extremely high").

2) To what extent do you agree with the claim that smoking in an older age (55 years old or above), significantly increases the risk of suffering from complications of the COVID-19 disease (severe illness, need for artificial respiration, death)?
_____________________ (from 1 representing "totally disagree" to 5 representing "fully agree").

3) In your opinion, to what extent does using threat appeals in anti-smoking advertisements can be effective for encouraging people to quit smoking? _________ (from 1 representing "extremely low" to 5 representing "extremely high").

4) In your opinion, to what extent does using COVID-19 as the threatening message in anti-smoking advertisements can be effective for encouraging people to quit smoking? _________ (from 1 representing "extremely low" to 5 representing "extremely high").

5) Do you think that using COVID-19 as the threatening message in anti-smoking advertisements is more effective for smokers who are 55 years old or above compared to young smokers (adults within the age range of 18-30 years old)? Yes/ No

Explanation:

____

____

____

____

____

6) Do you think that among young smokers (adults within the age range of 18-30 years old), using threat appeals, such as impotence and pregnancy risks in anti-smoking advertisements, is more effective than using COVID-19 as the threatening message? Yes/ No

Explanation:

____

____

____

____

____

____

Thank you for your cooperation,
Research team.

Figure S2: Illustration of the research process.

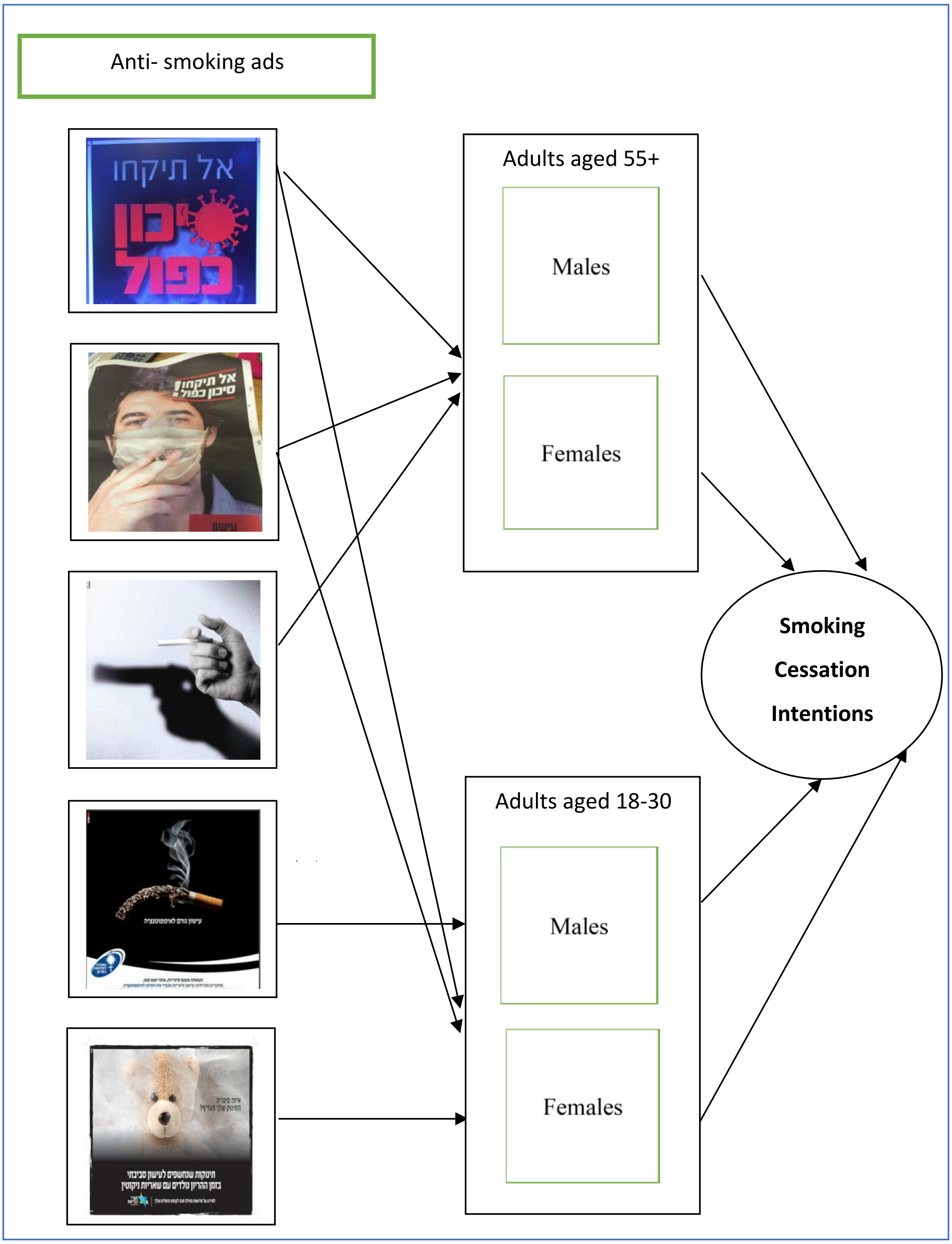

The ads' descriptions in Figure S1 are as follows (from top to bottom):

Ad 1) The ad includes a COVID-19 symbol and a threat: Do not take a double risk. In the bottom: "Smoking can cause a severe COVID-19 disease".

Ad 2) The ad includes a face mask and a threat: Do not take a double risk. In the bottom: "Smoking can cause a severe COVID-19 disease".

Ad 3) The ad includes a 'gunpoint' threat.

Ad 4) The ad includes the writings: "Smoking causes impotence" and in the bottom: " When you smoke cigarettes, you come out small".

Ad 5) The ad includes the writings: "Which cigarette does your baby prefer?" and in the bottom: "Babies who are exposed to environmental smoking during pregnancy are born with nicotine leftovers".

Supplementary Material S3: The Questionnaire

A Study Concerning Smoking

This questionnaire is part of a study by researchers from an academic institution.
Following is an anti-smoking advertisement. After looking at it you will be asked to fill in a questionnaire:

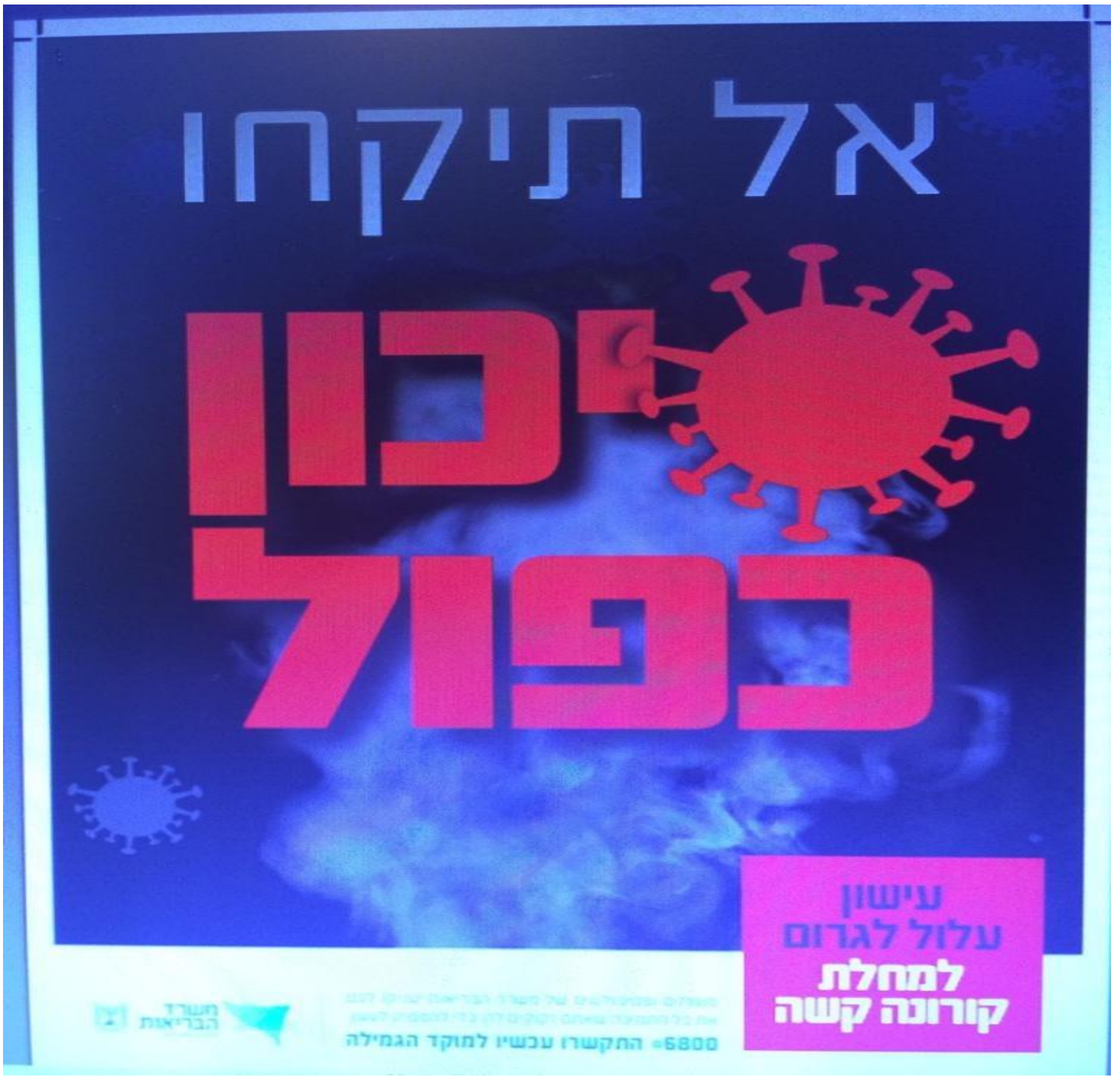

[The ad includes a COVID-19 symbol and a threat: Do not take a double risk.
In the purple rectangle: smoking can cause a severe COVID-19 disease].

In the following part of the questionnaire, please choose the answer that most accurately describes to what extent you feel that the ad you saw influenced you on a scale from 1 to 5:

	Definitely will not				Definitely will
1. How likely is it that in the next 3 months you will quit smoking completely and permanently?	1	2	3	4	5
2. How likely is it that in the next 3 months you will reduce the number of cigarettes a day you smoke?	1	2	3	4	5
3. How likely is it that in the next 3 months you will talk to someone (friend, family member, spouse) about quitting smoking?	1	2	3	4	5

	Not at all				Very much
1. Overall, how much did the ad make you worry about your health risks because of your smoking?	1	2	3	4	5
2. Overall, how much did the ad make you think about the serious health effects of smoking?	1	2	3	4	5
3. Overall, how much did the ad make you feel afraid or fearful about your smoking?	1	2	3	4	5

Attitude Questions

The following are some statements concerning smoking. Please indicate whether or not you agree with each of them on a scale from 1-totally disagree to 5- totally agree.

	Totally disagree				Totally agree
1. Smoking is extremely dangerous to my health	1	2	3	4	5
2. Smoking is ruining my health	1	2	3	4	5
3. My cigarette smoke leaves an unpleasant smell	1	2	3	4	5
4. Smoking gives me very bad breath	1	2	3	4	5
5. I spend too much money on cigarettes	1	2	3	4	5
6. My cigarette smoke bothers other people a great deal	1	2	3	4	5
7. My second-hand smoke is dangerous to those around me	1	2	3	4	5
8. Smoking is bad for my skin	1	2	3	4	5
9. It bothers me to be dependent on cigarettes	1	2	3	4	5
10. I would have more energy if I did not smoke	1	2	3	4	5
11. A cigarette calms me down when I am stressed	1	2	3	4	5
12. Smoking calms me down when I am upset	1	2	3	4	5
13. A cigarette helps me deal with difficult situations	1	2	3	4	5
14. After a cigarette, I am able to concentrate better	1	2	3	4	5
15. I like the motions of smoking	1	2	3	4	5
16. It feels so good to smoke!	1	2	3	4	5
17. I love smoking	1	2	3	4	5
18. I like to hold a cigarette between my fingers	1	2	3	4	5

Demographic Questions

1. Number of cigarettes smoked per day: ________
2. Age: _______
3. Gender:
   ( ) Male
   ( ) Female
4. The average net income per Israeli household is about 15,000 NIS. Your family's income is:
   ( ) Much below the average
   ( ) Below average
   ( ) Average
   ( ) Above average
   ( ) Much higher than the average
5. I am:
   ( ) Jewish
   ( ) Muslim
   ( ) Christian
   ( ) Other
6. I define myself:
   ( ) Secular
   ( ) Traditional
   ( ) Religious
   ( ) Orthodox
7. My family status is:
   ( ) Married
   ( ) Divorced
   ( ) Single parent
   ( ) Widower
   ( ) Bachelor
8. Number of children: ______
9. What is your education?
   ( ) Elementary or under
   ( ) High school without matriculation certificate
   ( ) High school with matriculation certificate
   ( ) B.A.
   ( ) M.A. or higher education
10. Have you or someone in your vicinity contracted the coronavirus (COVID-19)? Yes / No

Table S4: Summary of Experts' Opinions

	Expert #1	Expert #2	Expert #3	Expert #4	Expert #5	Expert #6	Expert #7	Expert #8
1. In your opinion, to what extent does smoking increase the severity of the COVID-19 disease, as well as the risk of suffering from complications? (from 1 representing "extremely low" to 5 representing "extremely high").	4	5	4	4	2	5	3	3
2. To what extent do you agree with the claim that smoking in an older age (55 years old or above), significantly increases the risk of suffering from complications of the COVID-19 disease (severe illness, need for artificial respiration, death)? (from 1 representing "totally disagree" to 5 representing "fully agree").	5	4	5	5	2	5	3	4
3. In your opinion, to what extent does using threat appeals in anti-smoking advertisements can be effective for encouraging people to quit smoking? (from 1 representing "extremely low" to 5 representing "extremely high").	3	1	4	2	1	2	2	2
4. In your opinion, to what extent does using COVID-19 as the threatening message in anti-smoking advertisements can be effective for encouraging people to quit smoking? (from 1 representing "extremely low" to 5 representing "extremely high").	4	4	5	2	2	2	4	4

	Expert #1	Expert #2	Expert #3	Expert #4	Expert #5	Expert #6	Expert #7	Expert #8
5. Do you think that using COVID-19 as the threatening message in anti-smoking advertisements is more effective for smokers who are 55 years old or above compared to young smokers (adults within the age range of 18-30 years old)? Yes/ No	Yes	Yes	Yes	Does not know	Does not know	Yes	Yes	Yes
6. Do you think that among young smokers (adults within the age range of 18-30 years old), using threat appeals, such as impotence and pregnancy risks in anti-smoking advertisements, is more effective than using COVID-19 as the threatening message? Yes/ No	No	Yes	No	Yes	Yes	Yes	Does not know	No

Supplementary Material S5: Experts' Opinions - Statements

Expert number 1:

Regarding question number 5: "The older adults (above 50) are more aware of the health risks in general. In my opinion, any threat appeal in an anti-smoking ad is more effective for older people. Young people are less afraid in general. They believe that nothing will happen to them".

Regarding question number 6: "Nowadays the issue of the coronavirus has a significant impact on everyone. As long as the crisis remains, I think that even the younger adults (18-30) will be more affected by ads that use the COVID-19 as the threatening message than any other threat appeal. As soon as the crisis will ebb away, I believe they will go back to being more afraid of the other risks (impotence and pregnancy complications)."

Expert number 2:

Regarding question number 5: "Youngsters believe that the COVID-19 is considered a disease with minor symptoms for the younger population, so they don't believe that they will suffer any harm from the disease. However, the older adults (55+) see that their peers suffer very severe illness or even death, and that their chances of surviving the disease are slim. Therefore, using COVID-19 as the threatening message in anti-smoking ads is more effective for smokers who are 55 years old or above, in the hope of reducing the risks of the disease."

Regarding question number 6: "Youngsters are afraid of impotence, whereas the older adults (55+) are afraid of COVID-19 from my experience."

Expert number 3:

Regarding question number 5: "It is common knowledge that the coronavirus is more severe among the older population than among the younger population. The older the individuals, the more they are cautious and frightened in general. Therefore, they are more afraid of COVID-19."

Regarding question number 6: "Although youngsters are extremely frightened by impotence, they are also very scared of the coronavirus. Recent studies, which were published in the media, showed that the virus causes a reduction in the male fertility. That can frighten the younger adults in general and the Jewish orthodox in particular."

Expert number 4:

Regarding question number 6: "Unfortunately, youngsters are not afraid of the coronavirus at all. On the other hand, the issues of impotence and pregnancy risks are very important to them. Hence, using impotence and pregnancy risks as the threatening message in anti-smoking ads is more effective for the youngsters than other threat appeal advertisements".

Personal comment:" Finally, from my perspective, even if the association to the COVID-19 will encourage only a few individuals to quit smoking, it is worthwhile."

Expert number 5:

Regarding question number 6: "Pregnant women are very anxious of the negative effects of smoking, so using pregnancy risks as the threatening message in anti-smoking ads is more effective. The threat of impotence is less effective from my experience."

Expert number 6:

Regarding question number 5: "In my opinion using COVID-19 as the threatening message in anti-smoking ads will definitely not be effective for youngsters. It may have a slight effect on individuals who are 55 years old or above, since they are considered a risk group."

Regarding question number 6: " From my experience**,** telling young people that if they smoke, they will need oxygen, a wheelchair, etc., will not influence them to try to quit smoking. For them it is considered a distant future. On the other hand, young men are very stressed about the risk of suffering from impotence, and young women are very anxious in regard to pregnancy complications."

Expert number 7:

Regarding question number 5: "There is greater awareness to the risk of suffering from any disease among the older population. Specifically, they are more aware of the risks inflicted upon them due to the coronavirus".

Regarding question number 6: "A warning about the higher risks associated with smoking and COVID-19 isn't more frightening to young people than warnings about cigarettes causing impotence or pregnancy complications. I am not sure which of the warnings will be more effective, but I assume there won't be a significant difference."

Expert number 8:

Regarding question number 5: "Young people perceive themselves as immune to illnesses. They believe that the health damages from smoking occur 'in their neighbor's home'. Most of them don't really care about their health. As people grow older their awareness to diseases increases. They hear about their peers getting sick or even dying of COVID-19 and empathize with them. Therefore, they understand the risks of smoking, which is why using COVID-19 as the threatening message in anti-smoking ads is more effective for smokers at the ages of 55 or above."

Regarding question number 6: "In spite of the above, in my opinion, everyone is afraid of the coronavirus nowadays. COVID-19 is in the core of the media and social discourse. Concerning the risk of impotence, I think that the youngsters believe that the risk does not apply to them. The same goes for pregnancy complications- young women who get pregnant easily, perceive the threat as farthest from them. Since the media gives global and local statistics regarding COVID-19 (including the number of deaths, number of patients that need artificial respiration, etc.), I think that using a COVID-19 warning in an anti-smoking ad would be more effective for individuals at any given age."

Table S6: Scales' Reliabilities (Cronbach's α)

Scales' Name	Reliability (Pre-Test)	Reliability (Main Study)
**Intentions to Quit Smoking**	0.82	0.82
**Perceived Threatening message**	0.93	0.94
**Attitude Towards Smoking**	0.83	0.80

Table S7: Scales' Names, Authors, and Items

Scale's Name	Authors	Items
**Perceived Threatening message**	Wong and Cappella (2009)	1. Overall, how much did the ad make you worry about your health risks because of your smoking?<br>2. Overall, how much did the ad make you think about the serious health effects of smoking?<br>3. Overall, how much did the ad make you feel afraid or fearful about your smoking?
**Attitude towards Smoking (ATS)**	Etter et al. (2000)	1. Smoking is extremely dangerous to my health.<br>2. Smoking is ruining my health.<br>3. My cigarette smoke leaves an unpleasant smell.<br>4. Smoking gives me very bad breath.<br>5. I spend too much money on cigarettes.<br>6. My cigarette smoke bothers other people a great deal.<br>7. My second-hand smoke is dangerous to those around me.<br>8. Smoking is bad for my skin.<br>9. It bothers me to be dependent on cigarettes.<br>10. I would have more energy if I did not smoke.<br>11. A cigarette calms me down when I am stressed.<br>12. Smoking calms me down when I am upset.<br>13. A cigarette helps me deal with difficult situations.<br>14. After a cigarette, I am able to concentrate better.<br>15. I like the motions of smoking.<br>16. It feels so good to smoke!<br>17. I love smoking.<br>18. I like to hold a cigarette between my fingers.
**Intentions to Quit Smoking**	Wong and Cappella (2009)	1. How likely is it that in the next 3 months you will quit smoking completely and permanently?<br>2. How likely is it that in the next 3 months you will reduce the number of cigarettes a day you smoke?<br>3. How likely is it that in the next 3 months you will talk to someone (friend, family member, spouse) about quitting smoking?

Table S8: The Main Study's Sub-groups

**Adults under 30 years old (N=418)**	**55 years old or above (N=303)**
Questionnaire included advertisements using COVID-19 fear appeal in the anti-smoking ad (**type 1**- COVID-19 symbol).	Questionnaire included advertisements using COVID-19 fear appeal in the anti-smoking ad (**type 1**- COVID-19 symbol).
Questionnaire included advertisements using COVID-19 fear appeal in the anti-smoking ad (**type 2**- face mask for preventing spreading COVID-19 and protecting yourself from COVID-19).	Questionnaire included advertisements using COVID-19 fear appeal in the anti-smoking ad (**type 2**- face mask for preventing spreading COVID-19 and protecting yourself from COVID-19).
Questionnaire included advertisements using other fear appeals in the anti-smoking ad (**for male participants**- threat of **impotence**).	Questionnaire included advertisements using other fear appeals in the anti-smoking ad (a **general threat** – a cigarette held in a way that is metaphoric to a **'gunpoint'**).
Questionnaire included advertisements using other fear appeals in the anti-smoking ad (**for female participants**- threat of **pregnancy risks**).	
